# Prenatal thyroid hormones accelerate postnatal growth and telomere shortening in wild great tits

**DOI:** 10.1242/jeb.243875

**Published:** 2023-03-27

**Authors:** Bin-Yan Hsu, Nina Cossin-Sevrin, Antoine Stier, Suvi Ruuskanen

**Affiliations:** ^1^ Department of Biology, University of Turku, FI-20014 Turku, Finland; ^2^ Université de Strasbourg, Centre National de la Recherche Scientifique, Institut Pluridisciplinaire Hubert Curien, UMR 7178, 67087 Strasbourg, France; ^3^ Univ Lyon, Université Claude Bernard Lyon 1, CNRS, ENTPE, UMR 5023 LEHNA, F-69622 Villeurbanne, France; ^4^ Department of Biological and Environmental Science, University of Jyväskylä, FI-40014 Jyväskylä, Finland

**Keywords:** Developmental programming, Thyroxine, Triiodothyronine, Ageing, Mitochondria, Metabolism, Oxidative stress, Maternal effects

## Abstract

The early-life environment is known to affect later-life health and disease, which could be mediated by the early-life programming of telomere length, a key hallmark of ageing. According to the fetal programming of telomere biology hypothesis, variation in prenatal exposure to hormones is likely to influence telomere length. Yet, the contribution of key metabolic hormones, i.e. thyroid hormones (THs), has been largely ignored. We recently showed that in contrast to predictions, exposure to elevated prenatal THs increased postnatal telomere length in wild collared flycatchers, but the generality of such effect, the underlying proximate mechanisms and consequences for survival have not been investigated. We therefore conducted a comprehensive study evaluating the impact of THs on potential drivers of telomere dynamics (growth, post-natal THs, mitochondria and oxidative stress), telomere length and medium-term survival using wild great tits as a model system. While prenatal THs did not significantly affect telomere length a week after hatching (i.e. day 7), they influenced postnatal telomere shortening (i.e. shorter telomeres at day 14 and the following winter) but not apparent survival. Circulating THs, mitochondrial density or oxidative stress biomarkers were not significantly influenced, whereas the TH-supplemented group showed accelerated growth, which may explain the observed delayed effect on telomeres. We discuss several alternative hypotheses that may explain the contrast with our previous findings in flycatchers. Given that shorter telomeres in early life tend to be carried until adulthood and are often associated with decreased survival prospects, the effects of prenatal THs on telomeres may have long-lasting effects on senescence.

## Teaser

**Summary:** Exposure to higher prenatal thyroid hormones can lead to accelerated cellular ageing in wild great tits, measured through telomere length.

## INTRODUCTION

Early-life environment has been repeatedly observed to affect adult health and survival prospects in human and non-human vertebrates (Barnes and Ozanne, 2011; Godfrey and Barker, 2001; Metcalfe and Monaghan, 2001). While the mechanisms underlying such delayed effects remained somewhat elusive (Barnes and Ozanne, 2011), the early-life programming of telomere length (i.e. the protective end caps of chromosomes) has emerged as a key candidate (Entringer et al., 2018). Telomere length is considered to be a hallmark of ageing (López-Otín et al., 2013) as telomeres shorten with age, and shorter telomeres are often predictive of lower survival or lifespan in both epidemiological and experimental studies (Arbeev et al., 2020; Heidinger et al., 2012; Muñoz-Lorente et al., 2019; Wilbourn et al., 2018). The prenatal hormonal environment, such as exposure to elevated glucocorticoid levels, has been identified as an important factor influencing early-life telomere length and its associated long-term outcomes (Criscuolo et al., 2020; Haussmann et al., 2012; Marchetto et al., 2016; Parolini et al., 2019). While there has been a considerable interest in prenatal glucocorticoids (Haussmann et al., 2012; Tissier, Williams, and Criscuolo, 2014; Noguera et al., 2022) and to a lesser extent androgens (Parolini et al., 2019; Tissier et al., 2014) in the context of the ‘fetal programming of telomere biology’ hypothesis (Entringer et al., 2018), the potential impact of prenatal thyroid hormones has been mostly ignored so far (Stier et al., 2020a).

Thyroid hormones (THs) are key coordinators of development and metabolism (McNabb, 2007), which are transferred from mothers to offspring (Ruuskanen and Hsu, 2018). Variation in exposure to prenatal thyroid hormones (T3, triiodothyronine, and T4, thyroxine) could influence telomere length via several mutually non-exclusive proximate pathways. (i) Prenatal THs can stimulate growth (although results are inconsistent across studies and species; Hsu et al., 2020, 2019a, 2017; Medici et al., 2013; Ruuskanen et al., 2016a; Sarraude et al., 2020a,b; Vrijkotte et al., 2017; Zhang et al., 2019), which can directly contribute to telomere attrition through increasing cellular division (Monaghan and Ozanne, 2018; Stier et al., 2020a,b), or indirectly accelerate telomere shortening through increasing oxidative stress (Monaghan and Ozanne, 2018; Reichert and Stier, 2017; Smith et al., 2016). (ii) Elevated TH levels are often associated with higher metabolic rates (Liu et al., 2006; Mullur et al., 2014; Welcker et al., 2013) and stimulate mitochondrial aerobic metabolism (Cioffi et al., 2013), both of which can potentially increase reactive oxygen species (ROS) and oxidative damage (Stier et al., 2014), accelerating telomere shortening (Reichert and Stier, 2017). It was recently shown that exposure to elevated prenatal TH levels can lead to a sex-specific increase in metabolic rate and circulating TH levels shortly after hatching (rock pigeons *Columba livia*, Hsu et al., 2017; but see Ruuskanen et al., 2016a), which suggests that prenatal hormones may programme postnatal metabolism and TH-axis function. (iii) The ‘metabolic telomere attrition hypothesis’ (Casagrande and Hau, 2019) postulates that telomere shortening might be adaptive by amplifying the cellular signalling of energy debt to re-direct critical resources to immediately important processes. In this scenario, we would expect accentuated shortening when catabolism is favoured over anabolism via mTOR inhibition (i.e. mechanistic target of rapamycin, a key regulator of cellular homeostasis), leading to a decrease in telomere maintenance processes and an active shortening through exonuclease action (Casagrande and Hau, 2019). As THs can have both anabolic and catabolic actions (Mullur et al., 2014), predictions can be made in both directions between prenatal THs and telomere length.

From an evolutionary perspective, increased offspring metabolism or growth diverts resources from somatic maintenance if resources are limited. This can accelerate damage to biomolecules and/or decrease repair/maintenance processes, and thus accentuate telomere shortening. Therefore, prenatal TH levels would be expected to vary in relation to predicted environmental conditions (as observed in terms of temperature and laying order, e.g. Ruuskanen et al., 2016b; Hsu et al., 2019b) to optimize this trade-off.

In contrast to most predictions relating prenatal THs to telomere length (see above), we recently reported that prenatal exposure to experimentally elevated THs increased telomere length in nestlings of a wild passerine, the collared flycatcher (*Ficedula albicollis*; Stier et al., 2020a). To better understand the potential generality of this surprising finding as well as assess underlying mechanisms and potential carry-over effects of variation in prenatal THs on later life-stages and survival, we conducted a more detailed study in another passerine species, the great tit (*Parus major*). The aim of this study was to comprehensively investigate the influence of prenatal THs on growth, oxidative stress, plasma THs, mitochondrial density (i.e. mitochondrial DNA copy number) and telomere dynamics as well as survival via an experimental manipulation of prenatal THs in a wild population. We monitored offspring multiple times during development and as juveniles a few months after fledging. Based on the majority of prior literature, we would predict that elevated prenatal THs would lead to faster growth, increased plasma THs, oxidative stress and mitochondrial density (higher density can lead to higher ROS production), ultimately accelerating telomere shortening. Alternatively, if our previous finding in the collared flycatcher reflected a general pattern, we would predict that despite accelerating growth, elevated prenatal THs would increase telomere length. We also would predict that elevated prenatal TH would increase post-fledging survival of the juveniles (e.g. as a consequence of both accelerated postnatal growth and potential beneficial effects for thermoregulation under low autumn–winter temperatures). However, longer-term survival, which we were not able to evaluate here, could be decreased, for example, as a result shorter telomeres.

## MATERIALS AND METHODS

The experiment and all methods we used were in accordance with all relevant guidelines and regulations and have been approved by the Animal Experiment Board of the Administrative Agency of South Finland (ESAVI/2902/2018) and the Environmental Centre of Southwestern Finland (licence number VARELY549/2018). The experiment was conducted in 2018 in a nest box population (314 nest boxes distributed over seven forest plots) on the island of Ruissalo in southwestern Finland (60°25′N, 22°10′E).

### Field experiment

The nest boxes were monitored with 5 day intervals to track egg laying. Yolk T3 and T4 levels were elevated in half of the nests by a combined injection of the two hormones into the egg (*n*=21 TH nests), following methods in Ruuskanen et al. (2016a). Control nests (*n*=21) were injected with the vehicle (0.9% NaCl) only. The TH content (mean±s.d.) in great tit eggs in the population was 0.053±0.02 ng yolk^−1^ for T3 and 0.46±0.16 ng yolk^−1^ for T4 (S.R., unpublished data). We aimed to raise the amount of yolk TH by 2 s.d. via injection into the egg yolk, a dose that has been recommended in relation to the natural hormone range of the study species (Podmokla et al., 2018). This corresponded to target doses of 0.041 ng yolk^−1^ for T3 and 0.325 ng yolk^−1^ for T4. To make sure injections would be performed on unincubated eggs mimicking maternal hormone levels (great tits can start incubation before the clutch is complete), injections were conducted on the day the 5th egg was laid to all eggs in the clutch. Thereafter, injections were conducted each day for the newly laid egg.

Hatching date and success were monitored by visiting nests daily starting before the estimated hatching day. Nestling body mass (∼0.01 g) and tarsus length (∼0.5 mm) were measured on day 2 (mass only), days 7 and 14 post-hatching. All nestling measurements were conducted between 08:00 h and 14:00 h. After measurements on day 2, nestlings were nail-coded, the brood was split and nestlings were cross-fostered with same-age nestlings in another nest of the opposite hormone treatment. This ‘split-brood design’ allows chicks from TH and control-injected eggs to be raised under the same postnatal environment and is in theory more sensitive to detect effects that may otherwise require a much larger number of nests in an experiment of ‘between-brood’ design (e.g. Tschirren et al., 2005). Preferentially, half of the brood was swapped between the nests with opposite treatments whenever possible, but we also split a brood for cross-fostering when we did not have an equal number of nests with opposite treatments on a given day. The proportion of TH nestlings (i.e. number of TH nestlings divided by the brood size) in each nest after cross-fostering was therefore recorded. All nestlings, cross-fostered or not, were included in the statistical analyses. There was no clear bias on the number of nestlings that were cross-fostered or stayed in the original nests with respect to the hormone treatment (control nestlings: 58 stayed and 41 moved, TH nestlings: 68 stayed and 51 moved, χ^2^=0.006, *P*=0.939). On day 7, a blood sample (∼40 µl) was taken and snap-frozen in liquid nitrogen for molecular analyses (telomere length, mitochondrial density and molecular sexing). On day 14, a small blood sample (10–15 µl) was snap-frozen in liquid nitrogen for oxidative stress biomarker analyses and stored at −80°C, while the rest of the sample (∼40 µl) was kept on ice, and centrifuged. Red blood cells (RBCs; 15 µl) were used for mitochondria and telomere measurements as above and plasma (15 µl) for TH analysis. After day 7 measurements, half of the nest were subject to a temperature manipulation (nest temperature increased for ∼2°C during day 7–day 14) for the purposes of another study. To avoid potential confounding effects, for data after day 7 and in juveniles, we only included the individuals from the nests which were not temperature manipulated (*n*=20 nests, 111 nestlings on day 7 and 99 nestlings on day 14). However, for day 2 and day 7 measurements (prior to the temperature manipulation), we prefer to include all data (*n*=42 nests, 224 nestlings in total, 218 nestlings on day 2 and 221 nestlings on day 7) to make use of the larger sample size.

To study long-term effects of prenatal thyroid hormones and offspring apparent post-fledging survival, we recaptured great tits during the following autumn–winter using mist-netting. Seven feeding stations were mounted in the study plots in August (generally any given nest box had a station within 200 m), and nets were positioned close to the feeding stations. The distance between adjacent feeding stations was ∼500 m, and most birds were captured at a different feeding station from their natal forest plot. In addition, 5 birds were captured at a constant effort site >3 km from the study plots, suggesting that all stations were potentially accessible to all birds. Each station was provided with ∼20 kg of peeled sunflower seeds and 2 kg of fat, checked and filled bi-weekly, and consumption was noted. Only in a very few cases were the feeders completely empty. Mist-netting was conducted at each feeding station for 3 h on 3 different days in September–October and similarly again in February, summing up to a total 126 h of mist-netting. The time of day (morning/midday/afternoon) was rotated for each site. Nets were checked every 30 min and mass (∼0.01 g) and wing length (∼0.5 mm) were recorded for each bird. A small blood sample (40–60 µl) was taken, kept on ice, centrifuged within 8 h, and RBCs frozen at −80°C for telomere and mitochondria density analyses.

### THs and oxidative stress assays

Plasma THs, the biologically active form T3 and a precursor T4 (expressed as ng ml^−1^) were measured in 14 day old nestlings with nano-LC-MS/MS following Ruuskanen et al. (2019, 2018). Because of practical constraints (time, effort and finances), we randomly selected 1–4 nestling per nest, *n*=13 TH and *n*=11 control nestlings (from 18 nests of origin and 11 nests of rearing) for analysis.

Total glutathione (tGSH), the most abundant intracellular antioxidant, and the ratio between reduced and oxidized glutathione (GSH:GSSG, an indicator of oxidative challenge) were measured from whole-blood samples (12 µl of 0.9% NaCl-diluted whole blood) with the ThioStar^®^ Glutathione Fluorescent Detection Kit (K005-FI, Arbor Assays, Ann Arbor, MI, USA) (Sarraude et al., 2020a). As a measure of oxidative damage, we assessed blood lipid peroxidation (malonaldehyde, MDA; 50 µl of 0.9% NaCl-diluted whole blood) using the TBARS assay following Espin et al. (2017). Both measurements had <10% coefficient of variation (CV) and are expressed per mg of protein (measured via BCA protein assay, ThermoFisher Scientific), following Espin et al. (2017). We aimed to measure biomarkers of oxidative stress from *n*=2 control or *n*=2 TH nestlings per nest, but sometimes fewer nestlings were available and some samples failed in the laboratory analysis, yielding the final sample size as *n*=33 TH and *n*=26 control nestlings from 18 rearing nests.

### qPCR assays for relative telomere length, mitochondrial DNA copy number and molecular sexing

We extracted DNA from blood cells using a standard salt extraction alcohol precipitation method (Aljanabi and Martinez, 1997). Extracted DNA was diluted in elution buffer BE for DNA preservation. DNA concentration and quality (*A*_260_/*A*_280_>1.80 and *A*_260_/*A*_230_>2.00) were checked with a ND-1000-Spectrophotometer (NanoDrop Technologies, Wilmington, NC, USA). DNA integrity was verified in 24 samples chosen randomly using gel electrophoresis (50 ng of DNA, 0.8% agarose gel at 100 mV for 60 min) and DNA staining with Midori Green. Each sample was then diluted to a concentration of 1.2 ng µl^−1^ for subsequent qPCR analysis.

Relative telomere length and mitochondrial DNA copy number (mtDNAcn, an index of mitochondrial density) were quantified using qPCR. This technique estimates relative telomere length by determining the ratio (*T*/*S*) of telomere repeat copy number (*T*) to a single copy gene (*S*), and the relative mtDNAcn as the ratio between one mitochondrial gene and the same single copy gene. Here, we used RAG1 as a single copy gene (verified as single copy using a BLAST analysis on the great tit genome) and cytochrome oxidase subunit 2 (COI2) as a mitochondrial gene (verified as non-duplicated in the nuclear genome using a BLAST analysis). Forward and reverse telomere primers were 5′-CGGTTTGTTTGGGTTTGGGTTTGGGTTTGGGTTTGGGTT-3′ (Tel-1b) and 5′-GGCTTGCCTTACCCTTACCCTTACCCTTACCCTTACCCT-3′ (Tel-2b), respectively. Forward and reverse RAG1 primers were 5′-TCGGCTAAACAGAGGTGTAAA-3′ and 5′-CAGCTTGGTGCTGAGATGTAT-3′, respectively. Forward and reverse COI2 primers were 5′-CAAAGATATCGGCACCCTCTAC-3′; 5′-GCCTAGTTCTGCACGGATAAG-3′, respectively. For the qPCR assays, the reactions were performed on a 384-QuantStudio^TM^ 12K Flex Real-Time PCR System (Thermo Fisher), in a total volume of 12 µl including 6 ng of DNA, primers at a final concentration of 300 nmol l^−1^ and 6 μl of SensiFAST™ SYBR lo-ROX (Bioline). Telomere, RAG1 and COI2 reactions were performed in triplicate on the same plates (10 plates in total); the qPCR conditions were: 3 min at 95°C, followed by 35 cycles of 10 s at 95°C, 15 s at 58°C and 10 s at 72°C. A DNA sample (a pool of DNA from 10 adult individuals) was used as a reference sample and was included in triplicate on every plate. The efficiency of each amplicon was estimated from a standard curve of the reference sample ranging from 1.5 to 24 ng. The mean reaction efficiencies were 109.1±1.8% for telomere, 102.2±1.6% for RAG1 and 96.3±1.1% for COI2. The relative telomere length and mtDNAcn of each sample were calculated as (1+Ef_Tel or COI2_)^ΔCqTel or COI2^/(1+Ef_RAG1_)^ΔCqRAG1^; where Ef is the amplicon efficiency and ΔCq is the difference in quantification cycle (Cq)-values between the reference sample and the focal sample. Intra-plate technical repeatability of telomere and mtDNAcn based on triplicates were 0.87 (95% confidence interval, CI [0.85–0.89]) and 0.96 [0.95–0.97], respectively. Inter-plate technical repeatability of telomere and mtDNAcn based on one repeated plate were 0.98 [0.97–0.99] and 0.77 [0.59–0.88], respectively.

The use of mtDNAcn as an index of mitochondrial density has been questioned in humans (Larsen et al., 2012), but we have previously shown good correlations between mtDNAcn and mitochondrial respiration rates in pied flycatcher (Stier et al., 2019). Great tits have quite peculiar telomeres, characterized notably by some ultra-long telomeres that do not seem to shorten with age in adults (Atema et al., 2019). As qPCR only provides an estimate of overall telomere length, it has been suggested it could be suboptimal for this study species. However, relative telomere length (i.e. measured using qPCR) in this species has been shown to shorten during the nestling stage (Stier et al., 2016, 2015), to respond to environmental factors (e.g. hatching asynchrony: Salmón et al., 2016; Stier et al., 2016, 2015) and to predict adult survival (Salmón et al., 2017). Within-individual repeatability of telomere length has recently been suggested to be an important factor to evaluate the pertinence of telomere length data in a given study/species (Kärkkäinen et al., 2022), and the biological repeatability in our dataset was *R*=0.66 [0.55–0.79], which is above the average reported by qPCR studies (i.e. *R*=0.47), and within the upper range of what has been reported for great tits (Kärkkäinen et al., 2022).

Birds were molecularly sexed using a qPCR approach (adapted from Chang et al., 2008; Ellegren and Fridolfsson, 1997). Forward and reverse sexing primers were 5′-CACTACAGGGAAAACTGTAC-3′ (2987F) and 5′-CCCCTTCAGGTTCTTTAAAA-3′ (3112R), respectively. qPCR reactions were performed in a total volume of 12 µl including 6 ng of DNA, primers at a final concentration of 800 nmol l^−1^ and 6 μl of SensiFAST^TM^ SYBR^®^ Lo-ROX Kit (Bioline). qPCR conditions were: 3 min at 95°C, followed by 40 cycles of 45 s at 95°C, 60 s at 52°C and 60 s at 72°C, then followed by a melting curve analysis (95°C 60 s, 45°C 50 s, increase to 95°C at 0.1°C s^−1^, 95°C 30 s). Samples were run in duplicate in a single plate and 6 adults of known sex were included as positive controls. Sex was determined by looking at the dissociation curve, with two peaks indicating the presence of a Z and W chromosome (female), and one peak indicating the presence of only the Z chromosomes (male).

### Statistical analyses

We ran several linear or generalized linear mixed models (LMMs/GLMMs) for both nestling and juvenile data. In order to account for genetic effects and the effects of growing environment other than prenatal hormones, we included the IDs of both the nest of origin (i.e. where a nestling hatched) and the nest of rearing (i.e. where a nestling grew up) as random intercepts in all suitable models. For physiological measurements, assay batch ID was included either as a random intercept or as fixed factors (depending on the number of levels, see Tables 1–3 for details). All initial models included the prenatal hormone treatment (TH versus control), date (continuous variable), cross-fostering (yes/no), brood size (continuous variable), proportion of TH nestlings (continuous variable) in a nest, and their interactions with hormone treatment as fixed factors. Except for hormone treatment, other variables were initially included because of their potential confounding effects on nestling growth and development but only retained in the final models when *P*<0.15 in order not to miss potentially significant factors. Nevertheless, in the final models, significance level was still set at 0.05. In models of nestling growth (i.e. body mass and tarsus length), the ID of each individual was included as a random intercept.

**Table 1.**
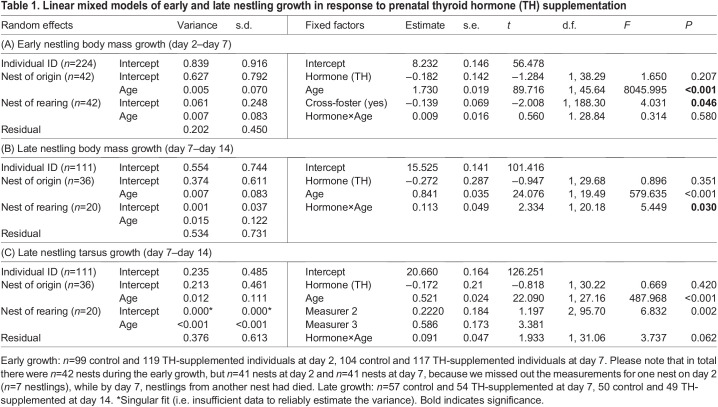
Linear mixed models of early and late nestling growth in response to prenatal thyroid hormone (TH) supplementation

To analyse nestling growth, we separated the data into the early (i.e. from day 2 to day 7) and late (i.e. from day 7 to day 14) phases. Because of the start of heating treatment on day 7, this separation allowed us to make use of the larger sample size during the early phase and focus on the nestlings from non-heated nests during the late phase. In these models, age (as a continuous variable) and the hormone×age interaction were included as additional fixed factors, and individual ID as a random intercept. In order to account for the variation in growth rate between nests, age (continuous variable) was also treated as a random slope to interact with both the original and rearing nests. For tarsus length, we additionally controlled for measurer ID as a fixed factor. Because molecular sexing was only conducted on nestlings that had DNA extracted for mtDNAcn and relative telomere length, not all nestlings included in the growth analyses were sexed. We therefore did not include sex in order to make use of the full data set. Repeating our models with only the sexed nestlings still gave qualitatively the same results and did not show sex-specific effects of prenatal THs on growth. For the juveniles captured during autumn/winter, we ran LMMs with hormone treatment, date of capture and sex (because of clear sexual size dimorphism at this age) as fixed factors.

For all models of physiological measurements, fixed factors included prenatal hormone treatment, nestling sex and body mass. If not showing any trends (i.e. *P*<0.15), sex and body mass were then removed from the final models, given the smaller sample sizes for the physiological measurements. Natural log transformation was conducted on highly skewed data – mtDNAcn and three oxidative stress biomarkers – to ensure normal residual distributions. Relative telomere length and plasma THs were untransformed as residuals qualified the assumption of normality. Because both mtDNAcn and relative telomere length are ratios to a single copy gene, we also *z*-transformed the data to allow across-study comparison (Verhulst, 2020). The ages of all juveniles were pooled as a single age category ‘autumn’, and age was thus included as a three-level categorical variable (day 7, day 14 and autumn) in order to estimate the changes of mtDNAcn and relative telomere length between each time point.

We used GLMMs to estimate the influence of prenatal THs on hatching success, fledging success (i.e. pre-fledging survival) and post-fledging survival. We modelled the outcome of each egg (i.e. hatched or not) or nestling (i.e. survived or not) using a logit link function, specifying a binomial residual distribution. For hatching success, two nests in which eggs never hatched (where the cause of failure was probably unrelated to our treatment) were excluded, giving a final sample size of 354 eggs from 42 nests. Nest ID was treated as a random intercept, and prenatal hormone treatment and laying date of each nest as fixed factors. For fledging success, we only included the 131 nestlings (reared in 27 nests after cross-fostering) that successfully hatched and were subsequently reared in the nests that were not subject to temperature manipulation in the analysis. Among the 131 nestlings, 99 successfully fledged and were included in the analysis of post-fledging survival. According to the nestling ringing records since 1999, only 8 out of >6200 birds ringed on Ruissalo Island were recaptured outside the island, suggesting a very low dispersal rate. The individuals that were never recaptured in the autumn were therefore presumed dead. The ID of rearing and original nests was treated as a random intercept and prenatal hormone treatment as the fixed factor. Laying date, brood size and cross-fostering, as well as their interactions with the hormone treatment, were initially included but only retained in the final model when *P*<0.15.

In all statistical models described above, hormone-related two-way interactions were of interest and therefore included. Following the suggestion by Schielzeth (2010), input variables (except for the technical variables) were mean-centred before model fitting. Significant interactions were further examined by *post hoc* interaction analysis using the R package emmeans (https://CRAN.R-project.org/package=emmeans).

All LMMs and GLMMs were conducted in the R environment 3.6.1, using the package lme4 (Bates et al., 2015) and lmerTest (Kuznetsova et al., 2017). *P*-values were determined using the Kenward–Roger method to approximate the denominator degrees of freedom (R package pbkrtest; Halekoh and Hojsgaard, 2014; implemented within lmerTest) for LMMs and by Laplace approximation for GLMMs. Model assumptions of normality and homoscedasticity were diagnosed by visual inspection on simulated residuals using the R package DHARMa (https://CRAN.R-project.org/package=DHARMa.). Clear violation was only observed in the models for plasma T3 in which residuals were heteroscedastic. Nevertheless, given the fact that the model of T3 did not detect any significant effect and the general robustness of LMMs (Schielzeth et al., 2020), this did not influence our conclusion.

## RESULTS

**Fig. 1.**
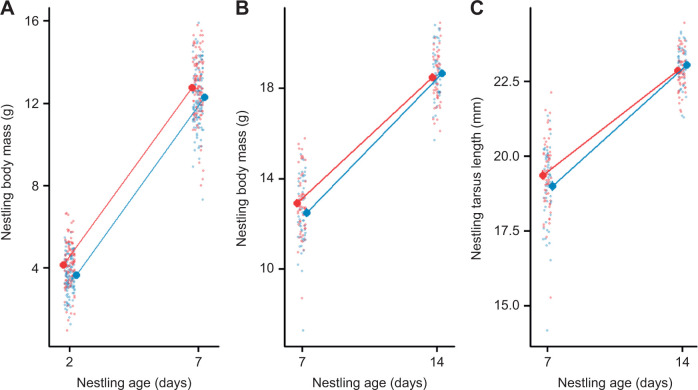
**Nestling growth in prenatally thyroid hormone (TH)-supplemented and control great tits.** (A) Body mass growth during the early nestling stage (day 2 to day 7 post-hatching). (B) Body mass growth in the late nestling stage (day 7 to day 14 post-hatching). (C) Structural size (tarsus length) growth during late nestling stages. Means±s.e.m. (large dots) and scatter of the raw data (small dots) are shown for TH-supplemented (blue) and control (red) groups. For sample sizes, see Materials and Methods.

Early body mass growth (between day 2 and day 7 post-hatching) did not differ between TH-supplemented and control groups (Table 1A, Fig. 1A), but individuals from TH-supplemented eggs grew faster at the later nestling stage, i.e. between day 7 and day 14 post-hatching (hormone×age interaction, *F*_1,20.18_=5.449, *P*=0.030, Table 1B; mean±s.e.m. body mass gain in TH nestlings 0.899±0.042 g day^−1^, control nestlings 0.786±0.043 g day^−1^, Fig. 1B). Similarly, individuals from TH-supplemented eggs expressed marginally faster tarsus growth rate between day 7 and day 14 post-hatching (*F*_1,31.06_=3.737, *P*=0.062, Table 1C; TH: 0.567±0.033 mm day^−1^, control: 0.476±0.034 mm day^−1^, Fig. 1C). Cross-fostering was retained in the final model of early body mass growth and suggested that fostered chicks had a lower growth rate (−0.139±0.069 g day^−1^) than non-fostered chicks (*P*=0.046, Table 1A). When analysing body mass and tarsus length separately for each age, body mass was slightly smaller on day 2 and day 7 in the TH-supplemented group compared with control, but there was no statistically significant difference between the groups at any age (Fig. 1; Table S1). At day 7, fostered nestlings tended to have a lower body mass by 0.347±0.179 g (*P*=0.053) on average, and had significantly shorter tarsi by 0.33±0.146 mm (*P*=0.025), although these differences disappeared by day 14 (Table S1). In juveniles, there was no difference in body mass or wing length between the treatment groups (body mass: *F*_1,9.79_=0.003, *P*=0.959; wing length: *F*_1,10.34_=0.011, *P*=0.919).

Biomarkers of intracellular oxidative status (tGSH, GSH:GSSG ratio) and damage to lipids 14 days post-hatching did not clearly differ among the treatment groups (marginal means±s.e.: tGSH: TH supplemented 0.253±0.022 µmol mg^−1^ protein, control 0.249±0.023 µmol mg^−1^ protein; GSH:GSSG ratio: TH supplemented 0.045±0.008, control 0.053±0.010; MDA: TH-supplemented 0.038±0.005 nmol mg^−1^ protein, control 0.037±0.005 nmol mg^−1^ protein; Table S2). Plasma T3 and T4 also did not differ among prenatally TH-supplemented and control groups 14 days post-hatching (marginal means±s.e. T3: TH supplemented 1.21±0.17 pmol ml^−1^, control 1.23±0.19 pmol ml^−1^; T4: TH supplemented 7.35±0.946 pmol ml^−1^, control 7.39±1.02 pmol ml^−1^; Table S3).

**Fig. 2.**
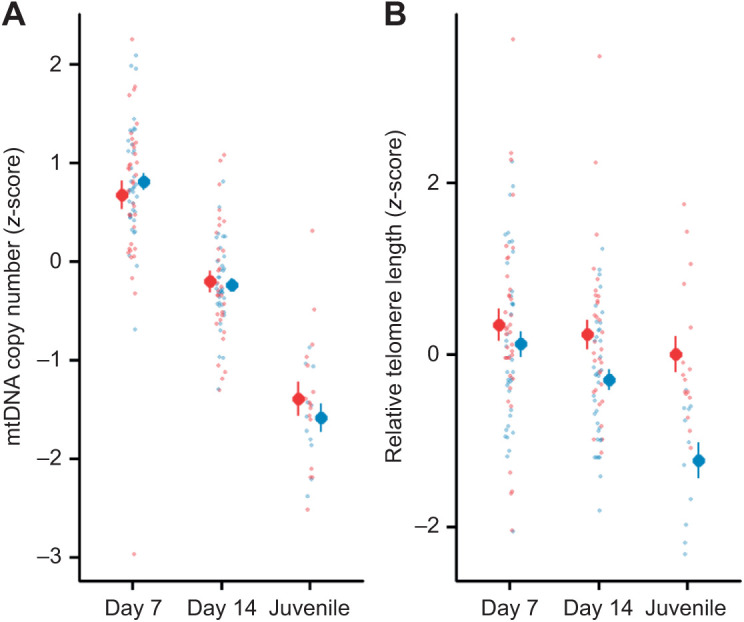
**Mitochondrial density and telomere length in TH-supplemented and control groups.** (A) Relative mitochondrial DNA (mtDNA) density and (B) relative telomere length in prenatally TH-supplemented (blue) and control groups (red) at different developmental stages (day 7 and 14 post-hatching and as juveniles, ∼3 months of age). Means±s.e.m. (large dots) and scatter of the raw data (small dots) are shown. For sample sizes, see Materials and Methods.

Mitochondrial density decreased with age, but there were no clear differences between prenatally TH-supplemented and control groups at any age (day 7, day 14 or juveniles; Fig. 2A, Table 2A). *Post hoc* analyses indicated a significant decrease between each age category (all Tukey-adjusted *P*<0.001), regardless of the hormone treatment.

**Table 2.**
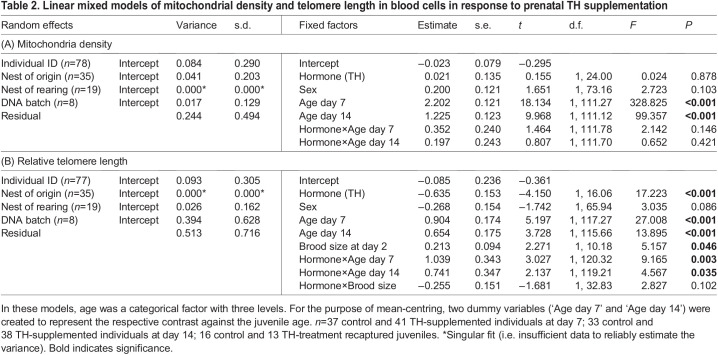
Linear mixed models of mitochondrial density and telomere length in blood cells in response to prenatal TH supplementation

Telomere length decreased with age (Table 2, Fig. 2B), but the pattern was different between the TH-supplemented and control group (Table 2, Fig. 2B). Early in the nestling phase, the difference between the prenatally TH-supplemented and control group approached significance (day 7, Tukey *post hoc* test: TH versus control: *t*_46.2_=1.804, *P*=0.078). On day 14 post-hatching, telomeres were significantly shorter (13%) in the TH group (Tukey *post hoc* test, TH versus control: *t*_46.8_=3.166, *P*=0.003), and this difference was accentuated in juveniles, with offspring from TH-supplemented eggs having telomeres that were ∼33% shorter than those of offspring from the control group (Tukey *post hoc* test, TH versus control: *t*_109.8_=4.311, *P*<0.0001). Further, we added the percentage of body mass gain from day 7 to day 14 in the model to examine whether the effect of prenatal TH supplementation on telomere length was via its effect on nestling growth, but this did not qualitatively change the results. The percentage of body mass gain did not show a clear association with telomere length (estimate±s.e.=−0.575±0.580, *F*_1,64.54_=0.981, *P*=0.326). Similarly, the three oxidative stress biomarkers also did not explain the difference in telomere shortening (all *P*>0.31).

Hatching success did not clearly differ among prenatally TH-supplemented and control individuals (TH 67.6%, control 69.1%; *z*=0.306, *P*=0.760; Table 3A). While nestling survival to fledging was higher in prenatally TH-supplemented nestlings than in control nestlings (TH: 83.05% versus control 69.44%), our model did not provide clear support for such a difference (*z*=0.136, *P*=0.892; Table 3B) and an analysis from egg to fledgling stage yielded qualitatively similar results (treatment *P*=0.86). Fledging success was lower in the nests with larger brood sizes (z=−2.333, *P*=0.020; Table 3B). Juvenile recapture rate was not significantly different across the groups (TH: 28.57% versus control 34.00%, *z*=−0.993, *P*=0.321; Table 3C).

**Table 3.**
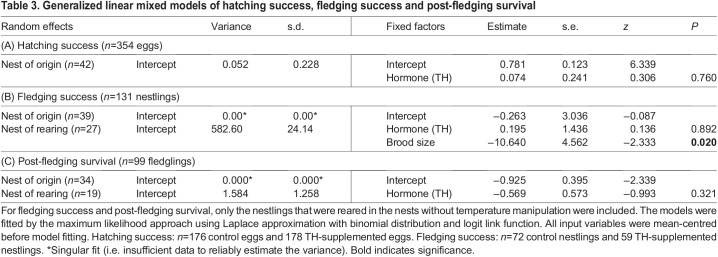
Generalized linear mixed models of hatching success, fledging success and post-fledging survival

## DISCUSSION

By manipulating prenatal exposure to THs in a wild passerine species, we demonstrate that an increase in prenatal THs can accelerate both postnatal growth and telomere shortening. Yet, we did not detect significant effects of elevated prenatal THs on postnatal oxidative stress levels, cellular energetics measured as mtDNA copy number, circulating TH levels or short- to medium-term survival (i.e. hatching success, fledging success and apparent survival to the next autumn/winter).

Shorter telomeres in the TH-supplemented group were only detected from 14 days after hatching onwards, which seems to exclude direct effects or prenatal TH effects on telomere dynamics during embryonic development. However, this coincides with the accelerated postnatal body mass and tarsus growth observed in the TH-supplemented group (between day 7 and 14) compared with controls, and faster growth can accelerate telomere shortening either through enhanced cell division or through inducing oxidative stress (Monaghan and Ozanne, 2018). Considering the lack of impact of prenatal THs on oxidative stress reported here and in previous work on birds (Hsu et al., 2020, 2019a; Sarraude et al., 2020a,b), it is not surprising that the accelerated telomere shortening observed in this study could not be explained by the measured oxidative stress biomarkers. Yet, we cannot fully rule out this explanation as DNA damage was not directly assessed and oxidative stress sensitivity might vary between different biomolecules (Reichert and Stier, 2017). The effect of prenatal THs on telomere length seemed to increase with age (i.e. stronger effect observed in juveniles than at day 14), which could be explained by a delayed effect of fast postnatal growth (day 7 to day 14) because telomeres only shorten at the subsequent cellular division, and therefore delayed effects are likely to be observed (Monaghan and Ozanne, 2018). Nevertheless, including body mass growth between day 7 and day 14 in the model did not explain the difference in telomere shortening. Our hypothesis that prenatal THs could programme postnatal metabolic and endocrine function (and thus affect telomere shortening indirectly) was not supported as we found no evidence for differences in mitochondrial density or plasma TH levels (key coordinators of metabolism) across the treatment groups. This result is not that surprising considering the limited evidence supporting a prenatal programming of plasma THs (sex-specific effect on T4 only: Hsu et al., 2017; no effect: Hsu et al., 2020) or mitochondrial density (no effect: Stier et al., 2020a; Hsu et al., 2020). However, our measurements are limited to blood, and tissue-specific effects of TH on mitochondrial density or TH-related cellular machinery (receptors and converting enzymes; Ruuskanen et al., 2022) may occur as this has been shown for instance for glucocorticoids (Weber et al., 2002). According to the metabolic telomere attrition hypothesis, telomere shortening is likely to be increased during energy-demanding periods, and accelerated growth under limited resources probably carries a metabolic cost (Casagrande and Hau, 2019).

The effects observed here on telomeres in response to increased prenatal TH levels are in sharp contrast to our previous findings in another passerine species, the collared flycatcher (Stier et al., 2020a). Indeed, increasing prenatal THs in the collared flycatcher increased telomere length measured very shortly after hatching (day 2), while not affecting telomere shortening during postnatal growth (Stier et al., 2020a). There are several alternative explanations for these contrasting findings: (1) we cannot exclude that prenatal THs increased telomere length during embryonic development in great tits as our first telomere length measurement (day 7, ∼70% of fledging body mass) was done considerably later than in collared flycatchers (i.e. day 2, ∼20% of fledging body mass). (2) THs differently influenced post-natal growth in the two species: great tits from the TH-supplemented eggs were initially slightly smaller, but grew faster in the late nestling period, whereas collared flycatchers from TH-supplemented eggs were bigger soon after hatching, but grew slightly slower during the nestling stage (Hsu et al., 2019a). These contrasting growth patterns may explain, at least partly, the contrasted findings in these two species: an increased metabolic demand for fast postnatal growth in great tits in response to prenatal TH could accelerate telomere shortening, while a reduced metabolic demand in collared flycatchers from TH-injected eggs could enable telomere maintenance (Casagrande and Hau, 2019). Measurements of the mTOR signalling pathway could shed light on the validity of this hypothesis (Casagrande and Hau, 2019). (3) The effects of maternal signals on offspring are expected to differ based on environmental conditions, so-called context-dependent effects; for example, prenatal androgens have been found to differentially influence offspring growth depending on season or food availability (Groothuis et al., 2020; Muriel et al., 2015). We recently found no evidence for prenatal THs differentially influencing growth and early-life survival depending on rearing temperature (Hsu et al., 2020), but the influence of resource availability was not tested. In two sister species, collared and pied flycatchers, differential effects of prenatal THs on growth may be explained by differences in food resources (Sarraude et al., 2020b). (4) These two species exhibit different life histories, as collared flycatchers are migratory and great tits are (relatively) sedentary. Migratory species have generally higher metabolism (Jetz et al., 2008) as well as an overall faster pace of life (Soriano-Redondo et al., 2021), and may thus present differences in TH physiology. We speculate that telomere maintenance and the role of THs in the regulation of telomere maintenance may differ across species with different life histories. Telomere maintenance is known to be influenced by, for example, species lifespan (Haussmann et al., 2004) and migratory populations within a species have shorter telomeres (Bauer et al., 2016). However, to our knowledge, studies have not considered migratory versus non-migratory species in telomere maintenance. Species-specific effects of the prenatal hormonal environment on telomere dynamics have already been described: elevated prenatal glucocorticoid levels led to shorter telomeres in domestic chicken and female zebra finch (Haussmann et al., 2012; Tissier et al., 2014), but to longer telomeres in yellow-legged gull (Noguera et al., 2022). To understand whether any of the hypotheses presented above are likely to explain the contrasted effects found here and in Stier et al. (2020a), more studies on the impact of prenatal THs on telomere biology across taxa are needed.

As mentioned above, in the collared flycatcher, elevated yolk THs increased nestling body mass at day 2 (Hsu et al., 2019a), whereas in the present study, elevated yolk THs only non-significantly reduced great tit nestling body mass at day 2 (marginal mean±s.e.=−0.402±0.245, *P*=0.109). In both studies, the lighter group of nestlings caught up with the other group before fledging. It therefore appears that the main difference lies in the differential effects of THs during the peri-hatching period. Interestingly, another experiment in the same population of great tits found that elevated yolk THs significantly shortened the time needed to hatch by 0.6 days on average (Cossin-Sevrin et al., 2022). Despite the shorter developmental time, the nestling body mass at day 2 was also found to be non-significant (Cossin-Sevrin et al., 2022). Thus, more information is required to clarify the exact peri-hatching effects of prenatal THs in altricial birds.

To understand the selective pressures on maternal allocation and signalling, it is important to characterize true fitness effects from development to survival and lifetime reproductive success. In contrast to our predictions, we found no effects of elevated prenatal TH supplementation on apparent survival or individual condition during the first autumn/winter. Unfortunately, longer-term effects on survival and reproductive success are very challenging to measure in such an experimental setting in the wild, as the recruitment to first breeding is usually very low (e.g. <10%; Radersma et al., 2015), which requires a very high number of nests to be manipulated, something often not feasible for both logistical and ethical reasons. Yet, long-term effects of accelerated early-life telomere shortening may lead to a decrease in longer-term survival and lifespan (Eastwood et al., 2019; Heidinger et al., 2012). The negative effects on later stages suggest that there need to be benefits of high prenatal THs for the offspring or mother, potentially during early-life stages, where selection can be strong. In this study, the elevated growth rates could increase offspring competitive abilities early in life, but the effects of prenatal THs on growth seem to be highly inconsistent across studies (Hsu et al., 2020, 2019a, 2017; Ruuskanen et al., 2016a; Sarraude et al., 2020a,b). While circulating THs are known to be associated with health and ageing in humans and mammalian models (Bano et al., 2017, 2019; Møllehave et al., 2020), our study is the first to show that exposure to higher prenatal THs can lead to accelerated cellular ageing measured through telomere length. This should stimulate further research using both epidemiological and experimental approaches across taxa to uncover the potential regulation of telomere biology by THs both prenatally and postnatally.

## Supplementary Material

10.1242/jexbio.243875_sup1 Supplementary information
