# Supplementary material for: Prenatal thyroid hormones accelerate postnatal growth and telomere shortening in wild great tits
Source: J Exp Biol. 2023 Mar 27;226(6):jeb243875. doi: 10.1242/jeb.243875 (PMC10112984; doi:10.1242/jeb.243875)
Supplement: Supplementary information [file jexbio-226-243875-s1.pdf]

Table S1. Linear mixed models (GLMM) on the effects of prenatal thyroid hormone elevation on nestling body mass and tarsus length at day 2, day 7, and day 14 post-hatching. The denominator degree of freedom (ddf) were approximated by the Kenward-Roger method to calculate p values.

**(a) Body mass at day 2 (n = 218 nestlings)**						
**Random effects:**		**Variance**	**Std. Dev.**			
**Nest of origin (n=41)**	Intercept	0.481	0.694			
**Residual**		0.625	0.791			
**Fixed factors**	**Estimate**	**SE**	**t**	**df**	**F**	**p**
**Intercept**	3.849	0.123	31.426			
**Hormone (TH)**	-0.402	0.245	-1.639	1, 38.69	2.686	0.109
Sample size: n = 99 control and 119 TH-supplemented						
The total sample size at day 2 (n =218 nestlings from 41 nests) was smaller than that at day 7 because we missed the day 2 for one nest (n = 7 nestlings)						
**(b) Body mass at day 7 (n = 221 nestlings)**						
**Random effects:**		**Variance**	**Std. Dev.**			
**Nest of origin (n=41)**	Intercept	0.944	0.972			
**Nest of rearing (n=41)**	Intercept	<0.001	<0.001			
**Residual**		1.482	1.217			
**Fixed factors**	**Estimate**	**SE**	**t**	**df**	**F**	**p**
**Intercept**	12.495	0.175	71.459			
**Hormone (TH)**	-0.367	0.353	-1.040	1, 38.93	1.081	0.305
**Cross-foster (yes)**	-0.347	0.179	-1.945	1, 179.22	3.782	0.053
**Brood size**	-0.141	0.072	-1.956	1, 25.71	3.828	0.061
**Hormone × Cross-foster**	0.668	0.368	1.813	1, 28.49	3.286	0.080
**(c) Tarsus length at day 7 (n = 221 nestlings)**						
**Random effects:**		**Variance**	**Std. Dev.**			
**Nest of origin (n=41)**	Intercept	0.4916	0.700			
**Nest of rearing (n=41)**	Intercept	0.000§	0.000§			
**Residual**		0.982	0.991			
**Fixed factors**	**Estimate**	**SE**	**t**	**df**	**F**	**p**
**Intercept**	18.940	0.205	92.592			
**Hormone (TH)**	-0.275	0.322	-0.855	1, 42.78	0.731	0.397
**Cross-foster (yes)**	-0.330	0.146	-2.268	1, 178.84	5.145	0.025
**Proportion of TH nestlings**	-0.297	0.482	-0.617	1, 54.90	0.381	0.540
**Measurer 2**	0.211	0.199	1.059	2, 204.00	3.357	0.037
**Measurer 3**	0.523	0.204	2.561			
**Hormone × proportion of TH nestlings**	-1.711	0.972	-1.762	1, 58.65	3.103	0.083

Sample size: n = 104 control and 117 TH-supplemented

§ Singular fit (i.e. insufficient data to reliably estimate the variance)

Journal of Experimental Biology • Supplementary information

Table S1 (cont.)

(d) Body mass at day 14 (n = 99 nestlings)						
**Random effects:**		**Variance**	**Std. Dev.**			
**Nest of origin (n=34)**	Intercept	0.310	0.557			
**Nest of rearing (n=19)**	Intercept	0.228	0.477			
**Residual**		0.801	0.895			
**Fixed factors**	**Estimate**	**SE**	**t**	**df**	**F**	**p**
**Intercept**	18.603	0.176	105.693			
**Hormone (TH)**	0.099	0.303	0.747	1, 22.45	0.130	0.722
**(e) Tarsus length at day 14 (n = 99 nestlings)**						
**Random effects:**		**Variance**	**Std. Dev.**			
**Nest of origin (n=34)**	Intercept	0.132	0.363			
**Nest of rearing (n=19)**	Intercept	0.000[§]	0.000[§]			
**Residual**		0.283	0.532			
**Fixed factors**	**Estimate**	**SE**	**t**	**df**	**F**	**p**
**Intercept**	23.199	0.238	97.313			
**Hormone (TH)**	0.157	0.175	0.899	1, 27.77	0.808	0.376
**Measurer 2**	-0.488	0.265	-1.845	2, 61.90	4.014	0.023
**Measurer 3**	-0.066	0.256	-0.258			

Sample sizes: n = 50 control and 49 TH-supplemented

[§] Singular fit (i.e. insufficient data to reliably estimate the variance)

Table S2. Linear mixed models of blood oxidative stress biomarkers in response to prenatal TH-supplementation.

**(a) total GSH µmol/mg protein (n = 58 nestlings)**						
**Random effects:**		**Variance**	**Std. Dev.**			
**Nest of origin (n=29)**	Intercept	0.031	0.177			
**Nest of rearing (n=18)**	Intercept	0.050	0.223			
**Residual**		0.044	0.210			
**Fixed factors**	**Estimate**	**SE**	**t**	**df**	**F**	**p**
**Intercept**	-1.492	0.114	-13.148			
**Hormone (TH)**	0.014	0.105	0.128	1, 15.46	0.016	0.900
**GSH assay batch**	0.221	0.145	1.526	1, 16.57	2.328	0.146
**(b) GSH:GSSG ratio (n = 58 nestlings)**						
**Random effects:**		**Variance**	**Std. Dev.**			
**Nest of origin (n=29)**	Intercept	0.210	0.458			
**Nest of rearing (n=18)**	Intercept	0.031	0.175			
**Residual**		0.134	0.367			
**Fixed factors**	**Estimate**	**SE**	**t**	**df**	**F**	**p**
**Intercept**	-3.106	0.181	-17.207			
**Hormone (TH)**	-0.171	0.243	-0.703	1, 16.75	0.456	0.509
**Body mass**	0.106	0.059	1.794	1, 41.88	2.986	0.091
**Proportion of TH-nestlings**	-0.173	0.462	-0.375	1, 21.30	0.124	0.728
**GSH assay batch**	0.133	0.237	0.561	1, 16.87	0.307	0.587
**Hormone × Proportion of TH-nestlings**	1.787	0.926	1.930	1, 19.81	3.553	0.074
**(c) MDA nmol/µg protein (n = 59 nestlings)**						
**Random effects:**		**Variance**	**Std. Dev.**			
**Nest of origin (n=30)**	Intercept	0.004	0.059			
**Nest of rearing (n=18)**	Intercept	0.004	0.060			
**TBARS batch (n = 6)**	Intercept	0.100	0.317			
**Residual**		0.039	0.198			
**Fixed factors**	**Estimate**	**SE**	**t**	**df**	**F**	**p**
**Intercept**	-3.286	0.135	-24.340			
**Hormone (TH)**	0.036	0.067	0.536	1, 16.86	0.287	0.599

Sample sizes:
total GSH and GSH:GSSG ratio, n = 26 control and 32 TH-supplemented
MDA, n = 26 control and 33 TH-supplemented

Journal of Experimental Biology • Supplementary information

Table S3. Linear mixed models of blood plasma thyroid hormones growth in response to prenatal TH-supplementation.

**(a) T3 pmol/ml (n = 24 nestlings)**						
**Random effects:**		**Variance**	**Std. Dev.**			
**Nest of origin (n=18)**	Intercept	0.054	0.232			
**Nest of rearing (n=11)**	Intercept	0.127	0.357			
**Residual**		0.132	0.363			
**Fixed factors**	**Estimate**	**SE**	**t**	**df**	**F**	**p**
**Intercept**	1.218	0.147	8.273			
**Hormone (TH)**	-0.026	0.202	-0.128	1, 7.37	0.016	0.902
**(b) T4 pmol/ml (n = 24 nestlings)**						
**Random effects:**		**Variance**	**Std. Dev.**			
**Nest of origin (n=18)**	Intercept	0.738	0.859			
**Nest of rearing (n=11)**	Intercept	2.934	1.713			
**Residual**		5.721	2.392			
**Fixed factors**	**Estimate**	**SE**	**t**	**df**	**F**	**p**
**Intercept**	7.452	0.758	9.837			
**Hormone (TH)**	-0.041	1.113	-0.037	1, 6.57	0.001	0.973
**Sex**	2.101	1.119	1.877	1, 14.99	2.532	0.132

Sample sizes: n = 11 control and 13 TH-supplemented

Journal of Experimental Biology • Supplementary information
